# Explaining the variation in ^137^Cs aggregated transfer factor for wild edible plants as a case study on Koshiabura (*Eleutherococcus sciadophylloides*) buds

**DOI:** 10.1038/s41598-023-41351-7

**Published:** 2023-08-29

**Authors:** Seiji Hayashi, Mirai Watanabe, Masami Kanao Koshikawa, Momo Takada, Seiichi Takechi, Mai Takagi, Masaru Sakai, Masanori Tamaoki

**Affiliations:** 1 Fukushima Regional Collaborative Research Center, National Institute for Environmental Studies, 10-2 Fukasaku, Miharu, Fukushima 963-7700 Japan; 2 National Institute for Environmental Studies, 16-2 Onogawa, Tsukuba, Ibaraki 305-8506 Japan; 3 National Institute of Advanced Industrial Science and Technology, 1‑1‑1 Higashi, Tsukuba, Ibaraki 305‑8567 Japan

**Keywords:** Plant sciences, Biogeochemistry, Environmental sciences

## Abstract

The aggregated transfer factor (T_ag_) is commonly used to represent the actual transfer of radiocesium from soil to wild edible plants, but the values have shown substantial variation since the Fukushima nuclear accident. To elucidate the factors causing this variation, we investigated the effects of spatial scale and vertical ^137^Cs distribution in the soil on the variation of T_ag_-^137^Cs values for one of the most severely contaminated wild edible plants, *Eleutherococcus sciadophylloides* Franch. et Sav. (Koshiabura). The variation in T_ag_-^137^Cs values was not reduced by direct measurement of ^137^Cs deposition in soil samples from the Koshiabura habitat, as a substitute for using spatially averaged airborne survey data at the administrative district scale. The ^137^Cs activity concentration in Koshiabura buds showed a significant positive correlation with the ^137^Cs inventories only in the organic horizon of soil from the Koshiabura habitat. The ratio of ^137^Cs inventories in the organic horizon to the total ^137^Cs deposition in soil exhibited substantial variation, especially in broad-leaved deciduous forests that Koshiabura primarily inhabits. This variation may be the cause of the wide range of T_ag_-^137^Cs values observed in Koshiabura buds when calculated from the total ^137^Cs deposition in soil.

## Introduction

In forested areas contaminated by past nuclear accidents, such as the Chernobyl disaster in 1986, radiocesium circulates through the ecosystem, resulting in long-term radioactive contamination of forest products, such as mushrooms and wild strawberries, as well as game animals^1–4^. Many areas contaminated with radioactivity from the Fukushima Daiichi Nuclear Power Plant (FDNPP) accident are forested areas of which most have not yet been decontaminated. Therefore, prolonged contamination of various forest products at Fukushima and other affected areas is currently a major concern for the social and economic recovery of the region^5^.

Wild edible plants, which are important forest products, are integral to the unique Japanese culture in mountainous areas (Satoyama); they are utilized not only as food for local residents, but also serve as a means of communication through sharing of the products among people^6^. Therefore, the prolonged contamination of wild edible plants has resulted in impairment of the enduring Satoyama culture. Although more than 10 years have elapsed since the FDNPP accident, the radiocesium concentrations in some wild edible plants still exceed the regulatory limit for commercial food distribution (100 Bq/kg fresh matter [FM]). Among these plants, the buds (specifically, young shoots with immature leaves emerging from the buds) of *Eleutherococcus sciadophylloides* Franch. et Sav. (Koshiabura), a deciduous broad-leaved subcanopy tree species widely distributed in temperate forests in Japan, exhibit exceptionally higher radiocesium concentrations compared with other wild edible plants^7,8^. Koshiabura often inhabits Satoyama areas, and commonly grows in sunny openings in forests and on forest margins. Most municipalities in Fukushima Prefecture have restricted or voluntarily prohibited the transport of its buds^9^. In addition, many municipalities in prefectures neighboring Fukushima Prefecture also have restricted the commercial distribution of Koshiabura buds; as a result, the restriction range is the largest among Japanese wild edible plants^9^.

Estimation of the potential ingestion dose by members of the public through consumption of contaminated edible wild plants is an important focus for radiation protection^10^. Such estimation is particularly helpful to improve the quality of life for local residents who wish to enjoy the collection and consumption of wild foods once again^11^. In this regard, the aggregated transfer factor (T_ag_) is widely used to represent the relationship between radionuclide concentrations in wild edible plants and the soil. This factor is defined as the radionuclide activity concentration in edible parts (Bq/kg) divided by radionuclide deposition in the soil (Bq/m^2^). The T_ag_ values for ^137^Cs (T_ag_-^137^Cs) in wild edible plants and mushrooms reported after previous nuclear disasters^1,2,12–14^ vary widely not only among species but also within a species. For example, the T_ag_-^137^Cs of Koshiabura buds varies in a range spanning one or two orders of magnitude (10^−4^ to 10^−2^)^11,15^. The broad variation in T_ag_-^137^Cs values may be strongly influenced by the procedure for calculating the dominator (i.e., ^137^Cs deposition in the soil). However, the reason for the broad variation in T_ag_-^137^Cs values among wild edible plants remains unclear.

With regard to publicly available radiocesium concentration data for wild edible plants, detailed information on their collection points is scarce in most cases. Moreover, detailed measurements for ^137^Cs deposition by collecting soil samples at each collection point is time-consuming and expensive. Therefore, the dominator for calculation of T_ag_-^137^Cs of wild edible plants has been determined at various spatial scales, such as the municipality or smaller administrative district levels, spatially averaged scales calculated based on an airborne monitoring survey^11,16^, point scales obtained from publicly available soil monitoring data at a point neighboring the collection area^17^, and from a combination of publicly available air dose rate and soil monitoring data^15^. However, the spatial effect on the variability of the dominator for T_ag_-^137^Cs has not been thoroughly assessed at various spatial scales, including the finest scale (i.e., point scale). Komatsu et al.^16^ reported that the accuracy of T_ag_-^137^Cs values for edible plants and mushrooms was improved at a finer spatial scale, as evidenced by a comparison of data at the municipality (coarser) and administrative district (finer) scales. In addition, the habitat preference of each wild edible plant species is species-specific, such as within a forest, the forest margin, and outside a forest, and thus spatially averaged ^137^Cs deposition data may result in a large error in T_ag_-^137^Cs calculations.

Each individual wild edible plant will form an unique root system to acquire nutrients in accordance with the growth environment. Previous studies have clarified that diversity in root systems enables a plant to acquire water and nutrients in forest and grassland ecosystems^18–20^. The variation in vertical distribution of root systems may give rise to a soil-depth dependency on the uptake of radiocesium as well as nutrients in plant species^21–23^. In addition, the already known vertical distribution of the ^137^Cs activity concentration, which decreases exponentially with depth in the mineral horizon^24^, would influence this dependence. Therefore, calculation of T_ag_-^137^Cs using the total ^137^Cs deposition in the soil profile may not necessarily be appropriate to evaluate ^137^Cs transfer from the soil to plants. Although the number of measurements was limited, Kiyono et al.^25^ reported that the ^137^Cs concentration in Koshiabura shoots with fully developed leaves showed a more strongly significant positive correlation with ^137^Cs inventory in the organic horizon than that in the total soil profile. Given that the activity concentration of ^137^Cs in the current year’s Koshiabura shoots decreases in accordance with growth^26^, it may also be necessary to confirm whether the soil depth dependence applies to the concentration of ^137^Cs in buds with immature leaves.

The objective of the present study was to clarify the specific factors responsible for variation in T_ag_-^137^Cs between Koshiabura buds at the edible stage and the soil, especially from two viewpoints: the spatial scale and the depth dependency of ^137^Cs deposition in the soil. Three spatial scales (municipality, administrative district, and sampling point) were used. In addition, the correlation between ^137^Cs activity concentration in Koshiabura buds and ^137^Cs deposition in each soil layer was examined to investigate the soil-depth dependency. The adequacy of methods for T_ag_-^137^Cs calculation for wild edible plants is discussed based on the present evaluations.

## Results and discussion

### Effect of spatial scale of ^137^Cs deposition data on T_ag_-^137^Cs of Koshiabura buds

#### ^137^Cs activity concentration in Koshiabura shoots at Iitate Village

**Figure 1:**
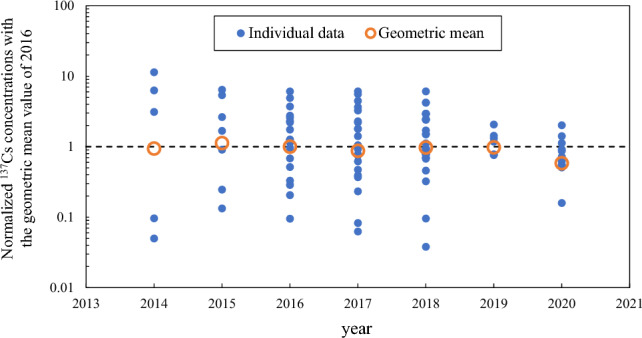
Annual change in ^137^Cs concentration in Koshiabura (*Eleutherococcus sciadophylloides*) buds reported by Iitate Village. The data are normalized in relation to the geometric mean value for 2016.

The ^137^Cs activity concentration (Bq/kg FM) in Koshiabura buds at Iitate Village have been measured since 2014 and published by the village office (Fig. 1). The ^137^Cs activity concentration data were converted using the geometric mean (GM) of 2016 and normalized to 1 to be easily comparable over time because of the relatively large sample number in 2016 (*N* = 20). The ^137^Cs activity concentrations in each year were used to derive the GM, geometric standard deviation (GSD), and maximum and minimum concentrations (Table S1). Although the number of samples is fixed to some extent in each year, which may help to increase the reliability of the average value, the data are for simple measurements conducted nondestructively and, therefore, the measurement error is likely to be large. The number of samples brought to the public inspection facility of the village office continued to rise until 2018, reflecting that the evacuation order was lifted in most areas of Iitate Village at the end of March, 2017, but the number appreciably decreased in 2019 and 2020. In addition, the variation in ^137^Cs activity concentration was smaller compared with that immediately after the lifting of the evacuation order. The reason for this is unclear, but it is speculated that local residents who have returned to the area may be hesitant to collect Koshiabura buds because the radiocesium concentrations continue to exceed the regulatory limit for commercial distribution (100 Bq/kg FM), despite the passage of time. Although the ^137^Cs activity concentration showed substantial variation in years other than the two mentioned, no significant increase or decrease in the average value was observed. This trend is consistent with previous studies that analyzed monitoring data for wild food products collected for self-consumption in other municipalities^15^.

**Figure 2:**
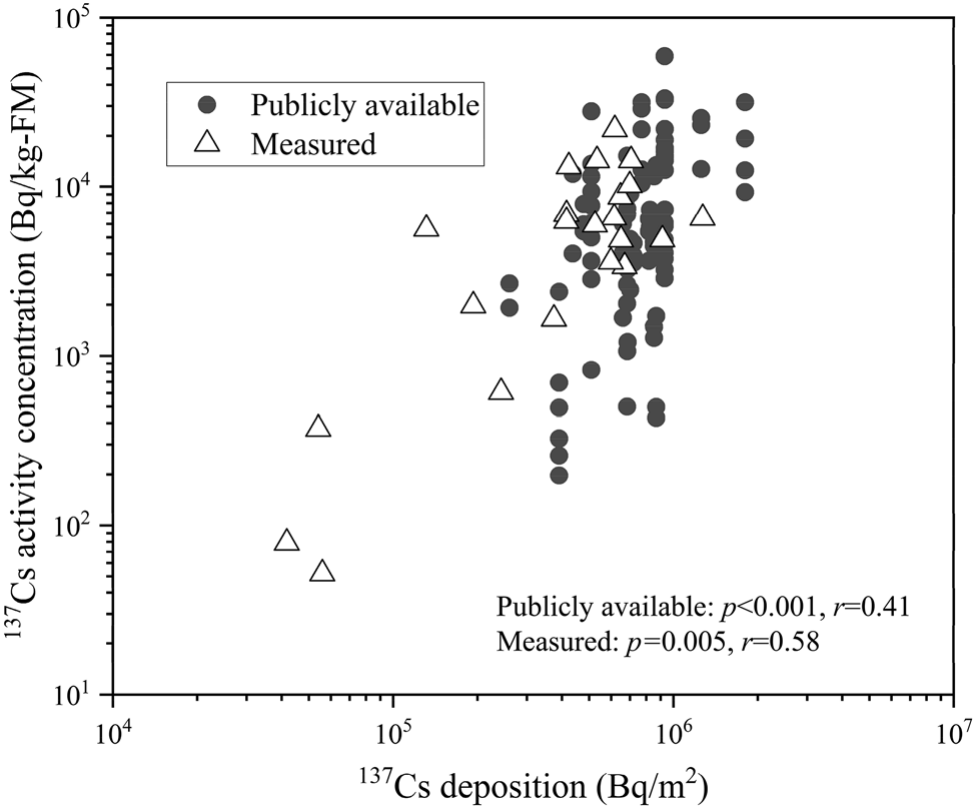
Relationship between ^137^Cs activity concentration in Koshiabura (*Eleutherococcus sciadophylloides*) buds and ^137^Cs deposition in soil of the plant’s habitat. Publicly available data were downloaded from the website of Iitate Village^49^.

Figure 2 shows the relationship between ^137^Cs activity concentration in Koshiabura buds and ^137^Cs deposition in soil. In the administrative district unit of Iitate Village, the ^137^Cs activity concentration in Koshiabura buds exhibited a trend to increase significantly with increase in average deposition of ^137^Cs in the soil, but this was accompanied by considerable variation (*p* < 0.001, *r* = 0.42). The measurement data at the point scale were also plotted in the graph and showed a significant positive correlation (*p* = 0.005, *r* = 0.58) and a similar trend to that for the administrative district unit.

#### Temporal change in T_ag_-^137^Cs of Koshiabura buds at Iitate Village

**Figure 3:**
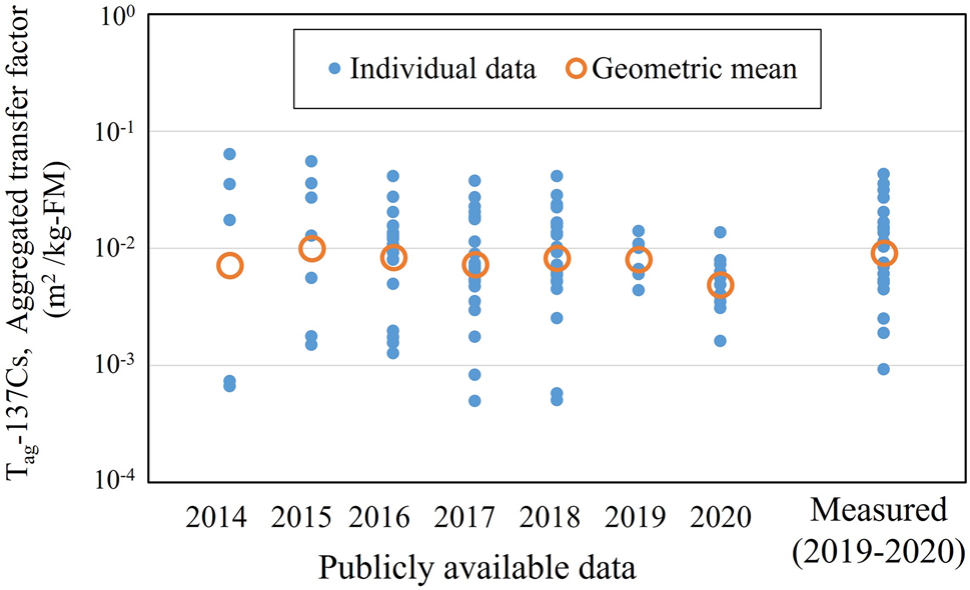
Annual change in ^137^Cs aggregated transfer factor (T_ag_-^137^Cs) of Koshiabura (*Eleutherococcus sciadophylloides*) buds calculated from data reported by Iitate Village and the spatially averaged ^137^Cs deposition in soil at the administrative district scale. The T_ag_-^137^Cs values calculated from direct measurements are also plotted.

The T_ag_-^137^Cs of Koshiabura buds calculated using averaged ^137^Cs deposition data per administrative district in each year showed substantial variability (Fig. 3), as did the ^137^Cs activity concentration in buds. This finding indicated that, even if Koshiabura buds were collected at sites with similar soil contamination or in the same district, broad variation in ^137^Cs activity concentration can be expected. With regard to interannual variation, similar to the results of analyses using publicly available data in other regions^11,15^, the annual GM values of T_ag_-^137^Cs showed no trend to significantly increase or decrease. Given that the data from one airborne monitoring survey on July 28, 2012^27^ (decay corrected to March 11, 2011) is used as the ^137^Cs deposition data for the calculation of T_ag_-^137^Cs, this “no trend” is the result of the strong influence of the trend of ^137^Cs activity concentration in Koshiabura buds shown in Fig. 1. Although the ^137^Cs activity concentration in Koshiabura buds was expected to decrease from year to year due to the progress of natural attenuation^28,29^ and fixation (aging)^30^ of ^137^Cs in the actual forest soil, this decrease was not observed. This suggests a stable and continuous transfer of radiocesium from the soil to the Koshiabura buds through the production and supply of a certain amount of bioavailable radiocesium.

#### Effect of spatial scale of ^137^Cs deposition data on T_ag_-^137^Cs of Koshiabura buds

Table 1 summarizes the GM, GSD, and maximum and minimum T_ag_-^137^Cs of Koshiabura buds reported in previous studies, including the present results. In each study, different methods were used to obtain ^137^Cs deposition data in soil at the Koshiabura bud collection sites, including spatial mean values at the municipal scale^11^ and administrative district scale^15^, and directly measured values at the collection site (the present study). Tagami et al.^15^ estimated spatially averaged ^137^Cs deposition in soil in each administrative district using a method that did not rely on spatially averaged values from airborne survey data. Instead, deposition data were calculated by applying an equation based on the relationship between measured soil inventory data and air dose rate, using air dose rate data measured in each administrative district at the time of Koshiabura buds collection in the municipality (https://emdb.jaea.go.jp/emdb/). In the present study, the publicly available data for Iitate Village (N = 90) were used for the calculation, covering the period after 2016, when the fluctuations in ^137^Cs activity concentration in buds were reduced.

**Table 1:** Aggregated transfer factor (m^2^/kg-FM) of ^137^Cs in Koshiabura (*Eleutherococcus sciadophylloides*) buds calculated using ^137^Cs deposition data for different spatial scales.

Spatial scale of ^137^Cs deposition data	*T*_*ag*_*-*^*137*^*Cs* (m^2^/kg-FM)				
	N	Geometoric mean	GSD	Min	Max
Municipalities^11^	22	5.2 × 10^−3^	3.5	5.4 × 10^−4^	5.3 × 10^−2^
Administrative districts^15^	16	7.3 × 10^−3^	2.0	1.3 × 10^−3^	3.2 × 10^−2^
Administrative districts (This study)	90	7.5 × 10^−3^	2.6	4.9 × 10^−4^	4.1 × 10^−2^
Local measurement (This study)	22	9.1 × 10^−3^	2.6	9.2 × 10^−4^	4.3 × 10^−2^

Approximate values of the ^137^Cs activity concentration in Koshiabura buds can potentially be estimated by understanding the ^137^Cs deposition status in the soil, regardless of the spatial scale. This is because the GM values are within the same order of 10^−3^ across all spatial scales. In addition, the variability in T_ag_-^137^Cs decreased with narrowing of the spatial scale, and the GSD decreased from 3.5 at the municipal scale to 2.0 or 2.6 at the administrative district scale. This finding was consistent with the results of Komatsu et al.^16^. Interestingly, the T_ag_-^137^Cs values calculated from directly measured data showed similar variability to those calculated from publicly available data for ^137^Cs activity concentration in Koshiabura buds and deposition in soil at the administrative district unit (GSD: 2.6 at local measurement scale vs. 2.0 or 2.6 at administrative district scale). Both the directly measured data and the publicly available data for ^137^Cs activity concentration in Koshiabura buds followed a log-normal distribution rather than a normal distribution. The directly measured ^137^Cs deposition in soil was the average of the measured data from samples collected at three sites near where the Koshiabura buds were collected. Although some variation in ^137^Cs deposition among the samples was evident, it was not necessarily large (the maximum, GM, and GSD of the coefficient of variation were 54%, 18%, and 1.7 among the 22 shoot collection sites, respectively). Therefore, the measured values were considered to adequately reflect the actual ^137^Cs deposition. In addition, differences in the water content ratio of Koshiabura buds, depending on the growth conditions at the collection site, might cause variability of T_ag_-^137^Cs through fluctuation in the ^137^Cs activity concentration determined on a fresh weight basis. The GM and GSD for T_ag_-^137^Cs using the dry-weight ^137^Cs activity concentration in the buds were calculated to be 5.6 × 10^−2^ (m^2^/kg) and 2.8, respectively. This resulted in slightly higher variability compared with when using the fresh-weight ^137^Cs activity concentration. These results suggested that a more accurate understanding of the total ^137^Cs deposition in soils of the Koshiabura habitat does not lead to elimination of T_ag_-^137^Cs variability. Thus, the overall soil contamination status is not necessarily directly reflected in the radiocesium concentration in Koshiabura buds.

### Relationship between ^137^Cs concentration in Koshiabura buds and vertical distribution of ^137^Cs deposition

**Figure 4:**
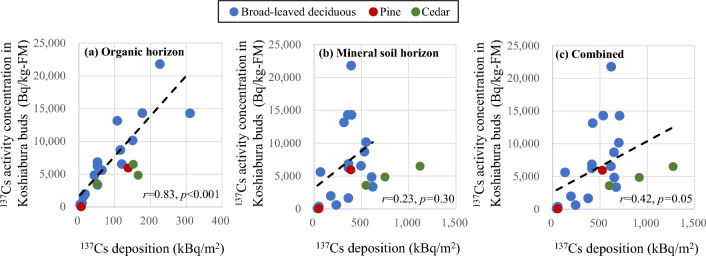
Relationship between ^137^Cs activity concentration in Koshiabura (*Eleutherococcus sciadophylloides*) buds and ^137^Cs deposition in the organic horizon (**a**), the mineral soil horizon (**b**), and the combined horizons (**c**). A regression analysis was conducted on the pooled data, including all forest types.

Figure 4a–c show the relationship between ^137^Cs activity concentration in Koshiabura buds (Bq/kg FM) and ^137^Cs deposition (Bq/m^2^) in the organic horizon, the mineral soil horizon (from the surface to 10 cm depth), and the combined horizons, respectively. Although Koshiabura buds were sampled from three different forest types, the regression analysis was conducted on the pooled data because most Koshiabura buds were sampled from broad-leaved deciduous forest. A strong correlation was observed between ^137^Cs inventories in the organic horizon and the ^137^Cs activity concentration in Koshiabura buds (*r* = 0.83, *p* < 0.001), which was consistent with previous studies^25,26^. However, for the mineral soil horizon, no significant positive correlation with ^137^Cs activity concentration in Koshiabura buds was observed, even though its deposition accounts for 51–97% of the total ^137^Cs soil deposition. Therefore, a weak positive correlation was found between the total ^137^Cs deposition in the soil and the ^137^Cs activity concentration in Koshiabura buds (*r* = 0.43, *p* = 0.05). This result strongly suggested that the amount of ^137^Cs inventory in the mineral soil horizon may not directly influence the differences in ^137^Cs activity concentration in Koshiabura buds among the sampling sites. Only for the samples collected from the cedar forest, the activity concentration in Koshiabura buds seemed to be positively related to the total deposition, although the number of samples was small. This probably resulted in the total ^137^Cs deposition in the soil reflecting the contamination level in the organic horizon in the cedar forest compared with that in the deciduous forest, as described later. However, further investigation with a larger number of samples is needed to clarify this.

With regard to the difference in contribution of the organic and mineral soil horizons to the increase in ^137^Cs activity concentration in Koshiabura buds, the shallow root system of Koshiabura probably has a strong effect, as pointed out in previous studies based on field observations^31,32^. The authors reported that the underground part of Koshiabura trees of height 0.5–2.0 m developed almost horizontally, and most of the main and lateral roots were distributed at a soil depth of 0–5 cm in the mineral soil horizon. While further research is needed to elucidate the mechanism, it is possible that dissolved ^137^Cs of high bioavailability, generated during forest litter decomposition, may be rapidly absorbed by Koshiabura roots in the surficial part of the mineral soil horizon. Consequently, the ^137^Cs activity concentration in Koshiabura buds may be a strong indicator of the contamination status of the organic matter accumulated in that horizon rather than that of the mineral soil horizon.

### Why does T_ag_-^137^Cs of Koshiabura buds vary largely?

As one important factor in the high variability of T_ag_-^137^Cs, the variation of soil properties in the mineral soil horizon should be considered, such as the radiocesium fixation ability of the soil and the cation composition of the soil solution, especially the concentrations of potassium and ammonium^33–35^. Although ^137^Cs fixation potentials were not measured at all four sampling sites in the present study, all of them have brown forest soils with granitic rocks as the surface geology^36^. Therefore, the ^137^Cs fixation potentials may be high^37^ (Yamaguchi et al.^38^) and have little influence on the among-site differences in root uptake by Koshiabura. Similarly, with regard to potassium and ammonium ions in the soil solution, although they were not measured in the present study, the concentration of ammonium ion is usually very low compared with that of potassium ions because of rapid nitrification by microorganisms at the surface of forest soils. Soil exchangeable potassium in the soil (mg K_2_O/100 g-ds), measured by extraction with 1 M ammonium acetate for 2 h, at all four sites ranged from 59 to 140 in the organic horizon and 16–89 in the 0–5 cm depth of the mineral soil horizon. Based on the effect of potassium fertilization on the reduction of radiocesium uptake by Japanese cypress seedlings grown in a stand contaminated by the FDNPP accident^38^, in addition to the abundance in the mineral soil horizon, the sufficient supply potential of potassium ions from the organic horizon at each site may not influence the control of ^137^Cs transfer via root uptake among the sites. Although more detailed investigation and analysis is required to draw a definitive conclusion, these results currently suggest that the soil properties in the mineral soil horizon are insufficiently variable to cause the large variation in T_ag_-^137^Cs at the sampling sites in the present study.

**Figure 5:**
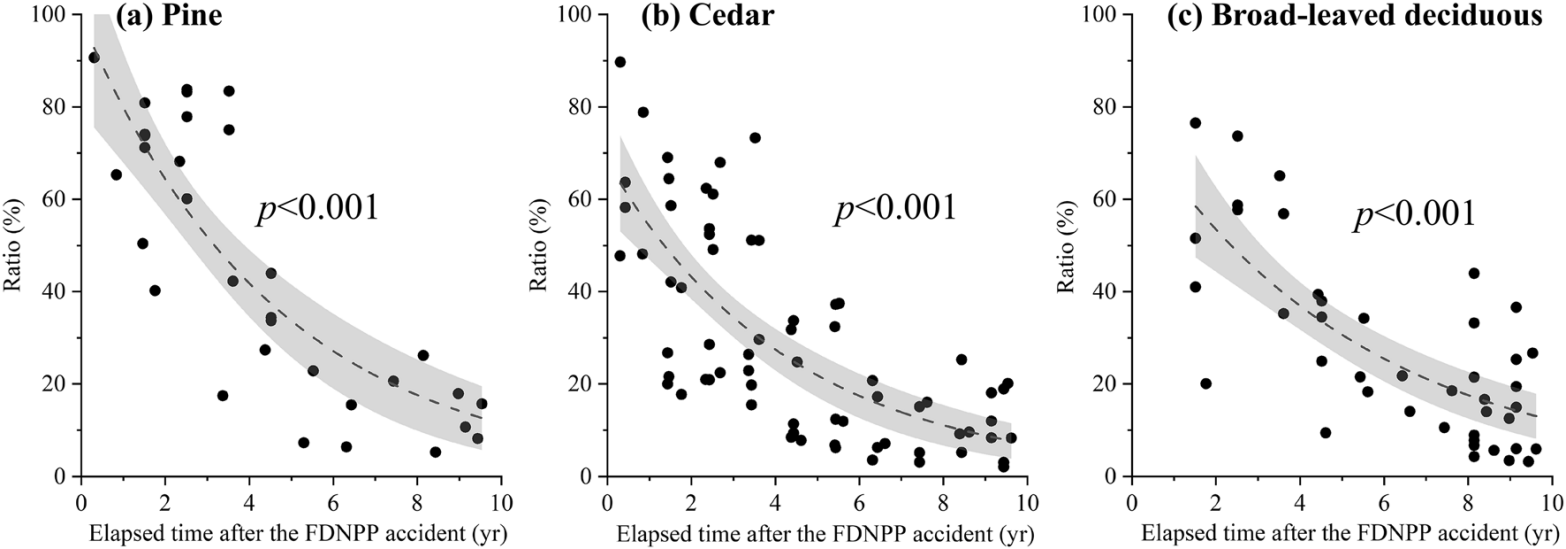
Temporal changes in the ratio of the total ^137^Cs deposition in forest soil retained in the organic horizon in pine, Japanese cedar, and broad-leaved deciduous forests after the FDNPP accident^24,28,39–43^. The dashed line depicts the approximate curve obtained by employing the exponential function. The range illustrated in gray signifies the 95% confidence interval.

The abundance of ^137^Cs in the organic horizon is also thought to be an important factor in the variation of T_ag_-^137^Cs, as shown by the significant positive correlation with ^137^Cs activity concentration in Koshiabura buds (Fig. 4a). Temporal changes in the ratio of ^137^Cs retained in the organic horizon to the total ^137^Cs deposition in the soil were summarized for each forest type based on the results of several previous studies and the present study (Fig. 5)^24,28,39–43^. The retention ratio has decreased significantly over time, regardless of the forest type. On average, the ratio may remain within a certain range (5–15%) with a slight decline in the future. However, broad-leaved deciduous forests, which are the primary habitat of Koshiabura, showed significantly greater variation in retention ratio (Fig. 5c) during the 8–10 years after the accident (i.e., our targeted period for calculating T_ag_-^137^Cs) than evergreen coniferous forests (Fig. 5a, b) (*F* test, *F* = 0.37, *p* = 0.04). Owing to differences in the ease of microbial degradation, radiocesium is more easily leached from deciduous hardwood litter than from cedar forest^44–46^. The transfer of radiocesium from the mineral soil horizon to the surface organic horizon through mycelial action (absorption) is more pronounced in broad-leaved deciduous forest than in cedar forest^47,48^. However, such microbial activities are ordinarily spatially heterogeneous and influenced by the environment on the forest floor. These factors are suggested to influence radiocesium accumulation in organic matter on the forest floor of broad-leaved deciduous forest, resulting in considerable variation in the ratio of ^137^Cs retained in the organic horizon relative to the total deposition in the forest soil. Given the strong positive correlation between ^137^Cs retention in the organic horizon and ^137^Cs activity concentration in Koshiabura buds, the substantial variation of the ratio in broad-leaved deciduous forests can cause broad variation in the ratio of ^137^Cs activity concentration in Koshiabura buds to the total ^137^Cs deposition in the soil (i.e., T_ag_-^137^Cs), even under similar degrees of contamination in the soil as a whole. This explains why there was no reduction in the variability of T_ag_-^137^Cs when using directly measured ^137^Cs deposition in soil at the point of shoot collection, compared with using spatial averages at the administrative district scale. If the variation in the ratio of radiocesium retained in the organic horizon relative to the total ^137^Cs deposition in soil is maintained in the future, understanding the actual status and trends in contamination of Koshiabura buds using T_ag_-^137^Cs will continue to entail considerable uncertainty.

The present results suggested that understanding the radioactive contamination status of the organic horizon may reduce the uncertainty in determining the ^137^Cs activity concentration of Koshiabura buds. For instance, a novel aggregated transfer factor for the relationship between ^137^Cs inventory in the organic horizon and the ^137^Cs activity concentration of Koshiabura buds could be proposed based on these findings. The value calculated in the present study was 0.075 ± 0.037 m^2^/kg. In addition, the ^137^Cs activity concentration in Koshiabura buds showed a strong significant positive correlation with the ^137^Cs concentration in the organic horizon (*r* = 0.80, *p* < 0.001). This indicated that the transfer factor, which represents the ratio of ^137^Cs concentrations in the buds and the organic horizon, can assist in determining the ^137^Cs activity concentration in Koshiabura buds. As considerable variation in T_ag_-^137^Cs has been reported for other wild edible plants^13^, it would be worthwhile to assess indicators that can more precisely indicate the transfer of ^137^Cs from the soil to wild edible plants, based on the relationship between the distribution of the root system of wild edible plants and the vertical distribution of ^137^Cs accumulation in the soil in the plant habitats.

## Methods

### Publicly available data collection from Iitate Village

**Figure 6:**
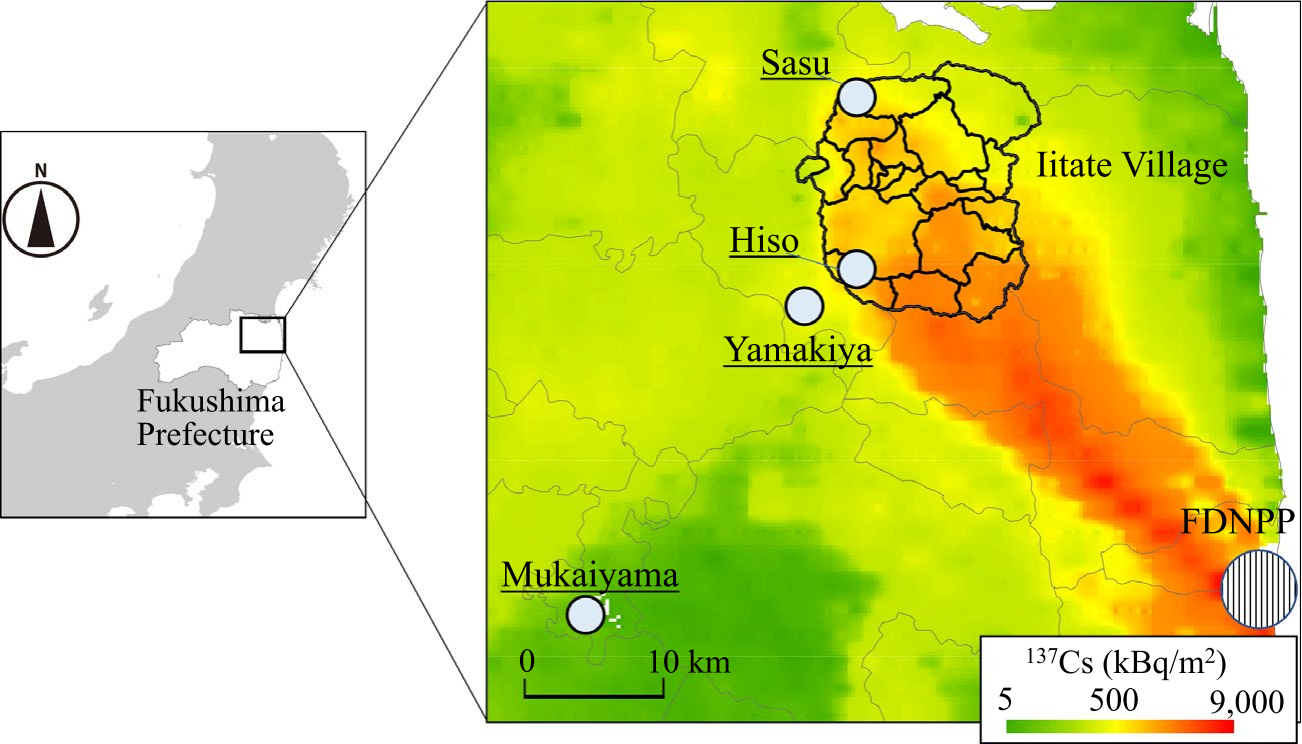
Location of Iitate Village split by administrative districts, and sampling sites of Koshiabura (*Eleutherococcus sciadophylloides*) buds and soil indicated on a map of ^137^Cs deposition amount generated from the fifth airborne survey by MEXT^27^. The statistical map data we purchased (ZENRIN CO., LTD., Tokyo, Japan, https://www.zenrin.co.jp/product/category/gis/contents/statistics/index.html) was used to draw the municipal division of Fukushima Prefecture and the administrative division of Iitate Village. The figure was drawn in ESRI ArcMap 10.8, which can be downloaded from the ESRI website (https://support.esri.com/en/Products/Desktop/arcgis-desktop/arcmap/10-8-2#downloads).

Data for the ^134^Cs and ^137^Cs activity concentration of Koshiabura buds with immature leaves, for each of 20 administrative districts, obtained from inspection of specimens brought by residents of Iitate Village, were used to evaluate T_ag_-^137^Cs variation in Koshiabura buds at a finer spatial scale than the municipality scale. The data were downloaded from the website of Iitate Village^49^. Iitate Village is located approximately 40–50 km northwest of the FDNPP (Fig. 6) and occupies an area of 230.1 km^2^ with a population of approximately 950 (6,211 in 2010 and 947 in 2022). The village is located in the center of the Abukuma Highlands, where the elevation ranges from 220 to 919 m above sea level. The area has forest coverage of 75.0%, which is higher than the average for Fukushima Prefecture (71%) and Japan as a whole (69%). Fukushima Prefecture established a system for each municipality to assess radioactivity in vegetables and mushrooms consumed by residents; Iitate Village started its inspection program during September, 2012. A simple inspection machine (Sonomamahakaru NDA2; Advanced Fusion Technology, Co., Ltd., Tokyo, Japan) with 5″ × 5″ NaI (Tl) scintillation detectors was installed at the eight public facilities. Inspections are conducted upon application by residents.

The measured ^134^Cs and ^137^Cs activity concentration data (Bq/kg FM) for Koshiabura buds have been reported since spring 2014. A total of 113 data sets had accumulated by the end of spring 2020. Of these, 102 data sets were used in the present analysis, excluding those for which ^134^Cs/^137^Cs ratios were far removed from 1.0 after decay correction on 11 March, 2011, and those for trees that were considered to have been transplanted from mountain forests and grown in home gardens based on field interviews.

The T_ag_-^137^Cs for Koshiabura buds was calculated on a FM basis (m^2^/kg FM) in the present study. Most T_ag_-^137^Cs estimates for forest products, such as trees, mushrooms, and berries, have been evaluated on a dry-weight basis in previous studies^25,50^ and in international reports^14,51^. The radioactivity concentrations in the aforementioned monitoring data sets were recorded on a FM basis (Bq/kg FM), but the water content of the samples was not recorded.

### Radiocesium deposition data from airborne survey

The same total radiocesium deposition data obtained from the results of the fifth airborne monitoring survey of 28 July, 2012, conducted by the Ministry of Education, Culture, Sports, Science and Technology (MEXT)^27^ was used to compare the results from our previous study in which T_ag_-^137^Cs of Koshiabura was calculated at the municipality scale^11^. As in several previous studies in forested areas after the FDNPP accident, a trend for a significant decline has not been detected in the deposition of ^137^Cs^24^^,^^42^, in addition to the very limited runoff from a forested catchment^52,53^. Thus, only one survey for deposition data was used for food monitoring data in 2014–2020.

A representative deposition value for each of 20 administrative districts in Iitate Village (Fig. 1) was statistically calculated using ArcGIS version 10.8 (ESRI, Inc., Redlands, CA, USA). The geometric mean value for the deposition densities was used as the representative value for each administrative district because the deposition densities in each administrative district showed log-normal distributions rather than normal distributions.

### Sample collection and pretreatment

To adequately compare the effect of the spatial scale of ^137^Cs deposition data on T_ag_-^137^Cs with publicly available data, Koshiabura buds in the same edible condition as those brought by local residents in Iitate Village and their habitat soil were collected during spring of 2019 and 2020 at four sites differing in radiocesium deposition level after obtaining permission from each of the forest owners (Fig. 6; 13 points in Sasu district and three points in Hiso district of Iitate Village, three points in Yamakiya district of Kawamata Town, and three points in Mukaiyama district of Miharu Town). The sampling dates are summarized in Table S1. The collected samples of Koshiabura buds were immediately transported to the laboratory and stored at 4 °C. After gently washing the samples with Milli-Q water to remove soil particles and absorbing surficial moisture with a disposable paper towel, the fresh mass was recorded. All samples were oven-dried at 60 °C for 48 h, the dry weight was recorded, and the samples were crushed. Each sample was mixed well and enclosed in a 100-ml plastic container for radioactivity measurement. Soil samples were collected at three points, representing the vertices of a regular triangle, situated 50 cm from a sampled Koshiabura tree. The organic matter within frames (700.7 cm^2^ in area) were sampled by hand. Fresh litter layers (L layers), fermentation layers, and humus layers (FH layers) were collected all together and defined as the organic horizon. After air-drying at 25 °C, the soil samples were passed through a 4-mm sieve after cutting litter that was not fragmented and fine roots with scissors, and tree bark fragments and taproots were removed by hand. The mineral soil within frames (353 cm^2^ in area) were sampled with a trowel, and collected sequentially from depths of 0–5 cm and 5–10 cm. After air-drying at 25 °C, the soil samples were passed through a 2-mm sieve.

### Radioactivity measurement

To convert the dry-weight activity concentration from the radioactivity measurements, a portion of the residue was oven-dried at 105 °C for 48 h and the moisture content was calculated.

The radioactivity of each sample was measured using a SEG-EMS GEM 35–70 coaxial high-purity germanium detector (Seiko EG&G, Tokyo, Japan) using Gammastudio software (Seiko EG&G). The radioisotope sources MX033U8PP (Japan Radioisotope Association, Tokyo, Japan) and EG-ML (Eckert & Ziegler Isotope Products, Valencia, CA, USA) were used to calibrate the system. Radioactivity was measured for as long as 100,000 s, depending on the radioactivity of the samples. The radiocesium activity concentration (Bq/kg FM) of the Koshiabura buds was derived on a FM basis from the ratio between the fresh and dry masses. Deposition densities of radiocesium (Bq/m^2^) were obtained by combining inventories in the organic and mineral soil horizons because the organic horizon still contained non-negligible amounts of radiocesium.

### Data analysis

The T_ag_ for ^137^Cs (T_ag_-^137^Cs) was calculated because ^137^Cs has a long half-life (T_1/2_ = 30.1 years) and therefore is suitable for long-term dose assessment. The ^137^Cs activity concentration for all samples and the ^137^Cs deposition data from the airborne survey were decay-corrected to March 11, 2011. The aggregated transfer factor (T_ag_) was calculated in accordance with IAEA-TRS 472^50^.

1 $$ {\text{T}}_{{{\text{ag}}}} -^{{{137}}} {\text{Cs }}\left( {{\text{m}}^{{2}} /{\text{kg FM}}} \right)\, = \,^{{{137}}} {\text{Cs }}\;{\text{in }}\;{\text{edible}}\;{\text{ part }}\left( {{\text{Bq}}/{\text{kg FM}}} \right)/^{{{137}}} {\text{Cs }}\;{\text{deposition }}\left( {{\text{Bq}}/{\text{m}}^{{2}} } \right). $$

As the T_ag_-^137^Cs values showed a log-normal distribution, Spearman’s rank correlation test was applied to examine the relationship between ^137^Cs deposition and ^137^Cs activity concentration of Koshiabura buds. An F-test was employed to assess the significance of the variations observed in the ratio of ^137^Cs retained in the organic horizon to the total ^137^Cs deposition in the soil across different forest types. These statistical analyses of the data were conducted using OriginPro version 2022b (OriginLab Corporation, Northampton, MA, USA). A significant relationship was determined at a probability level of 0.05.

### Ethical approval

We comply with relevant guidelines and legislation regarding the sample collection in the present study. The plant species, Koshiabura (*Eleutherococcus sciadophylloides*), in the present study is not endangered. The Koshiabura buds in 2019–2020 were collected in Iitate Village, Kawamata Town, and Miharu Town after obtaining permission from each of the forest owners and are stored in NIES in Miharu Town. Voucher specimens of plant materials in the present study do not exist.

## Summary

The focus of this study was to examine the specific factors responsible for variation in T_ag_-^137^Cs for Koshiabura buds, the wild edible plant most severely contaminated with radioactivity after the FDNPP accident. Two viewpoints were considered, namely, the spatial scale and depth dependency of ^137^Cs deposition in soil used for the calculation.

When using airborne survey data as the ^137^Cs deposition data in soil, the variation of T_ag_-^137^Cs values tended to be reduced by using more spatially limited average data at the administrative district scale rather than the municipal scale. However, when using the directly measured ^137^Cs deposition in soil, no reduction in variability compared with using the spatially averaged data at the administrative district scale was observed.

The relationship between the ^137^Cs activity concentration in Koshiabura buds and ^137^Cs deposition in each soil horizon of their habitat revealed that the inventory of ^137^Cs in the organic horizon showed a significant positive correlation with the activity concentration in the buds rather than the mineral horizon to a depth of 10 cm, which had a much higher ^137^Cs inventory. This is probably due to the high bioavailability of ^137^Cs in organic matter and the fine roots of Koshiabura are mostly distributed in the soil surface layer.

The marked variation in the ratio of ^137^Cs inventory in the organic horizon to the total soil deposition, especially in the broad-leaved deciduous forests that Koshiabura primarily inhabits, must be the cause of the broad variation in T_ag_-^137^Cs values of Koshiabura buds when calculated from the total ^137^Cs deposition in soil.

### Supplementary Information

Supplementary Table S1.

## Data Availability

The datasets generated during and/or analyzed during the current study are available from the corresponding authors on reasonable request.
