# Supplementary material for: Explaining the variation in 137Cs aggregated transfer factor for wild edible plants as a case study on Koshiabura (Eleutherococcus sciadophylloides) buds
Source: Sci Rep. 2023 Aug 29;13:14162. doi: 10.1038/s41598-023-41351-7 (PMC10465601; doi:10.1038/s41598-023-41351-7)
Supplement: Supplementary file 1 — Supplementary Table S1. [file 41598_2023_41351_MOESM1_ESM.pptx]

## Slide 1
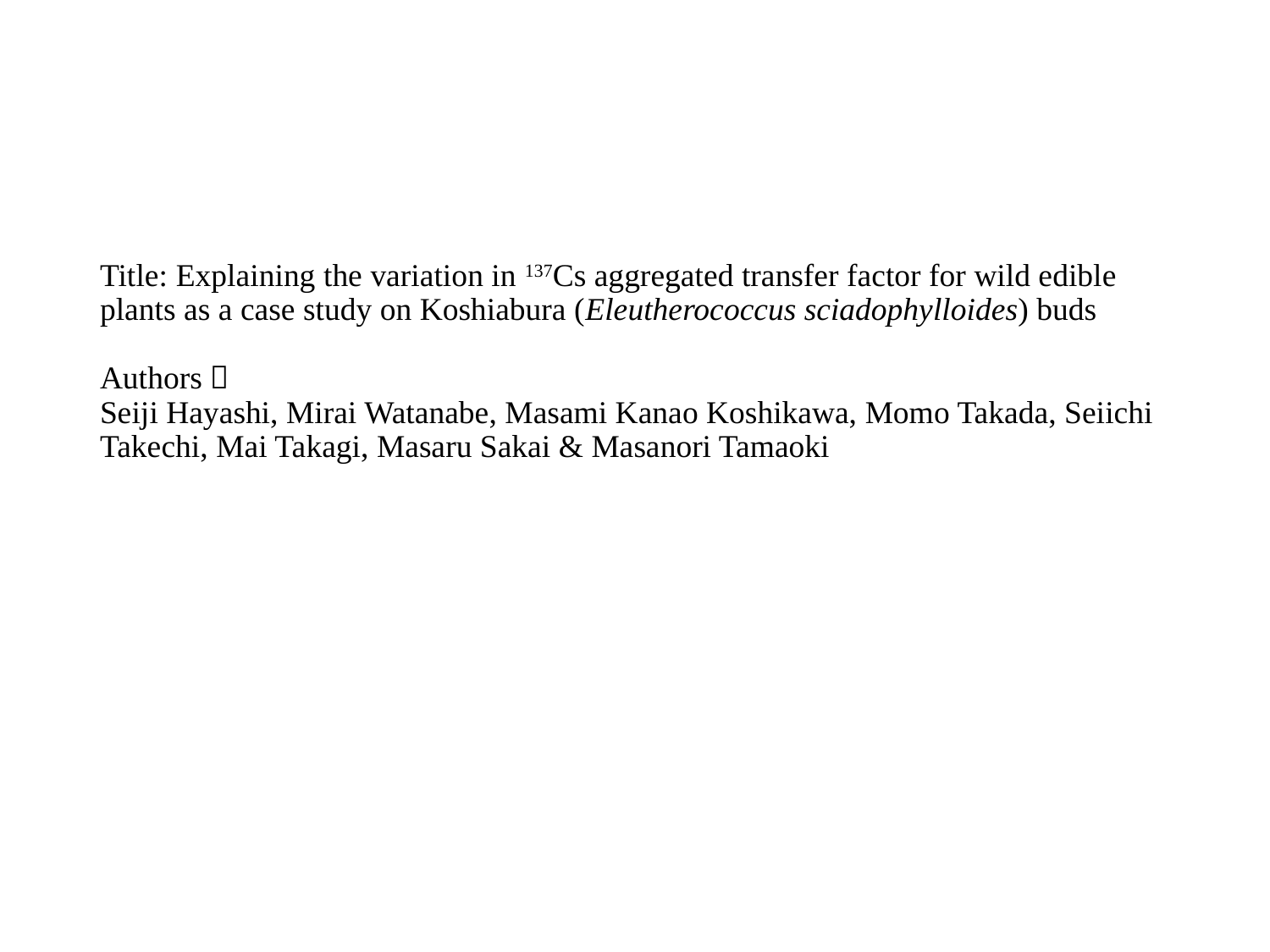

# Title: Explaining the variation in 137Cs aggregated transfer factor for wild edible plants as a case study on Koshiabura (Eleutherococcus sciadophylloides) buds Authors： Seiji Hayashi, Mirai Watanabe, Masami Kanao Koshikawa, Momo Takada, Seiichi Takechi, Mai Takagi, Masaru Sakai & Masanori Tamaoki

## Slide 2
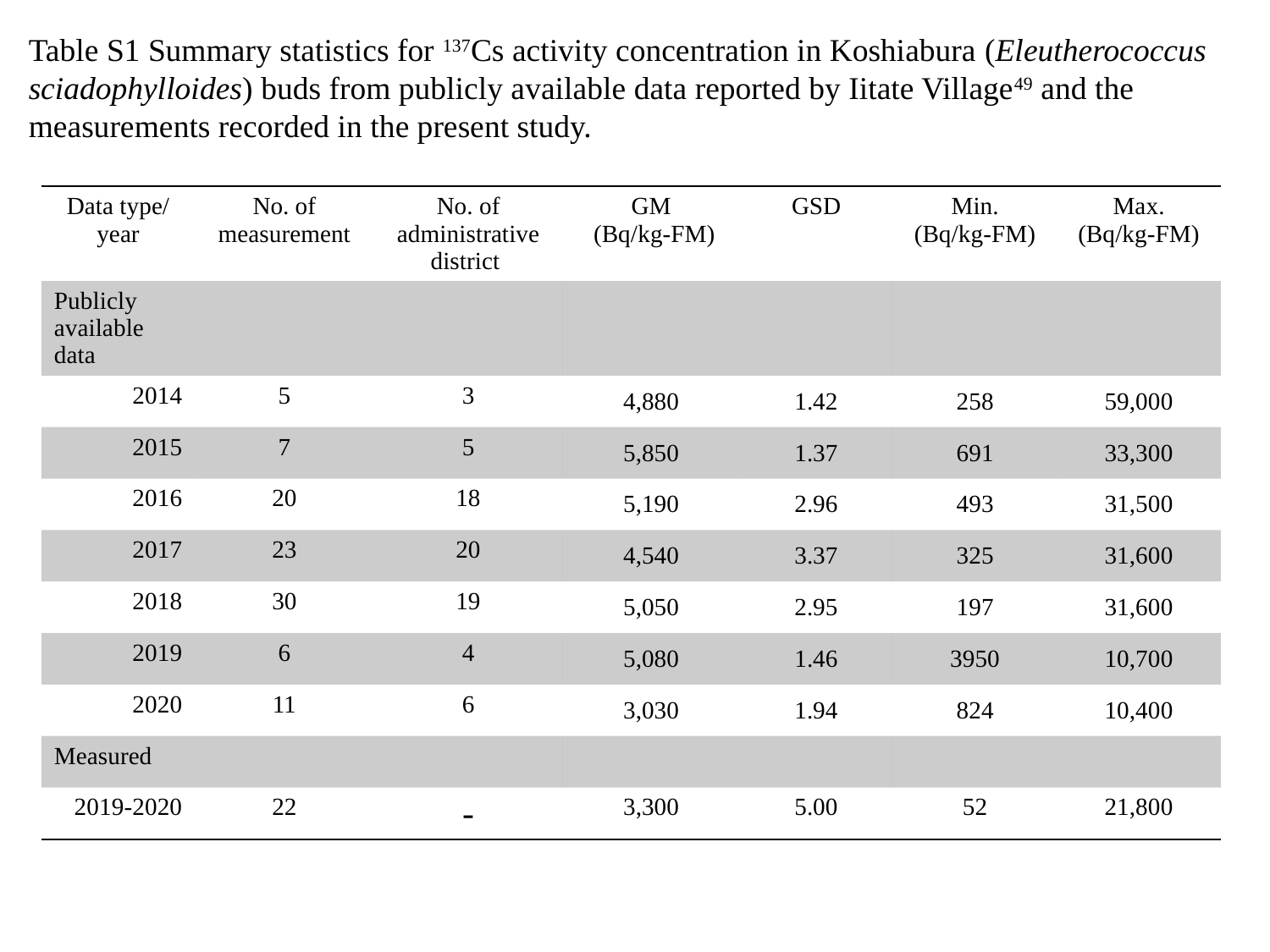

Table S1 Summary statistics for 137Cs activity concentration in Koshiabura (Eleutherococcus sciadophylloides) buds from publicly available data reported by Iitate Village49 and the measurements recorded in the present study.
| Data type/ year | No. of measurement | No. of administrative district | GM (Bq/kg-FM) | GSD | Min. (Bq/kg-FM) | Max. (Bq/kg-FM) |
| --- | --- | --- | --- | --- | --- | --- |
| Publicly available data | | | | | | |
| 2014 | 5 | 3 | 4,880 | 1.42 | 258 | 59,000 |
| 2015 | 7 | 5 | 5,850 | 1.37 | 691 | 33,300 |
| 2016 | 20 | 18 | 5,190 | 2.96 | 493 | 31,500 |
| 2017 | 23 | 20 | 4,540 | 3.37 | 325 | 31,600 |
| 2018 | 30 | 19 | 5,050 | 2.95 | 197 | 31,600 |
| 2019 | 6 | 4 | 5,080 | 1.46 | 3950 | 10,700 |
| 2020 | 11 | 6 | 3,030 | 1.94 | 824 | 10,400 |
| Measured | | | | | | |
| 2019-2020 | 22 | - | 3,300 | 5.00 | 52 | 21,800 |
